# Temporal trends and regional disparities in mortality from acute myocardial infarction in Brazil: a time series study, 2015-2024

**DOI:** 10.1590/S2237-96222026v35e20260533.en

**Published:** 2026-09-07

**Authors:** Geovanna Aires Silva, Carlos Henrique Santos, Jacqueline Andréia Bernardes Leão-Cordeiro, Antonio Marcio Teodoro Cordeiro Silva

**Affiliations:** 1 Pontifícia Universidade Católica de Goiás, Medicina, Goiânia, GO, Brazil Pontifícia Universidade Católica de Goiás Medicina Goiânia GO Brazil; 2 Universidade Federal de Goiás, Enfermagem, Goiânia, GO, Brazil Universidade Federal de Goiás Enfermagem Goiânia GO Brazil

**Keywords:** Cardiovascular Diseases, Aging, Health Status Disparities, Unified Health System, Time Series Studies, Enfermedades Cardiovasculares, Envejecimiento, Disparidades en el Estado de Salud, Sistema Único de Salud, Estudios de Series Temporales

## Abstract

**Objective:**

To analyze mortality from acute myocardial infarction in Brazil according to temporal trends, sociodemographic characteristics, and regional disparities from 2015 to 2024.

**Methods:**

A time series study using annual aggregated data from the Mortality Information System (*Sistema de Informações sobre Mortalidade*, SIM). Unadjusted mortality rates, age-standardized mortality rates, and sex-specific rate ratios were calculated. Temporal trends were assessed using log-linear regression to estimate the annual percent change (APC), and reestimated using the Prais-Winsten method as a complementary analysis.

**Results:**

A total of 938,997 deaths were recorded during the study period. The absolute number of deaths remained stable, with a 3.8% increase between 2015 and 2024. Age-standardized mortality rates declined throughout the study period (annual percent change: −2.6%; p-value<0.001). Mortality was higher among males, with a mean rate ratio of 1.53 over the study period, reaching 1.58 in 2024, and was concentrated among older adults. The Southeast and Northeast regions had the highest unadjusted mortality rates.

**Conclusion:**

Age-standardized mortality rates declined over the study period, although the high burden of deaths associated with population aging and regional disparities persisted. These findings reinforce the need to strengthen cardiovascular disease prevention strategies, timely diagnosis, and the organization of the healthcare network for cardiovascular diseases within the Brazilian Unified Health System (*Sistema Único de Saúde*, SUS).

## Resumen

**Objetivo:**

Analizar la mortalidad por infarto agudo de miocardio en Brasil según las tendencias temporales, las características sociodemográficas y las desigualdades regionales durante el período 2015-2024.

**Métodos:**

Estudio de series temporales con datos anuales agregados del Sistema de Información sobre Mortalidad (Sistema de Informações sobre Mortalidade, SIM). Se calcularon tasas brutas de mortalidad, tasas de mortalidad ajustadas por edad y razones de tasas de mortalidad por sexo. Las tendencias temporales se evaluaron mediante regresión log-lineal, con estimación del cambio porcentual anual (Annual Percent Change, APC), y se reestimaron mediante el método de Prais-Winsten como análisis complementario.

**Resultados:**

Se registraron 938.997 defunciones durante el período de estudio. El número absoluto de defunciones se mantuvo estable, con un incremento del 3,8% entre 2015 y 2024. Las tasas de mortalidad ajustadas por edad disminuyeron a lo largo de la serie temporal (cambio porcentual anual: −2,6%; valor de p < 0,001). La mortalidad fue mayor en los hombres, con una razón media de tasas de mortalidad de 1,53 durante el período analizado, que alcanzó 1,58 en 2024, y se concentró en los adultos mayores. Las regiones Sudeste y Nordeste presentaron las tasas brutas de mortalidad más elevadas. **Conclusión:** Se observó una disminución de las tasas de mortalidad ajustadas por edad durante el período analizado, aunque persistió una elevada carga de defunciones asociada al envejecimiento poblacional y a las desigualdades regionales. Estos hallazgos refuerzan la necesidad de fortalecer las acciones de prevención de las enfermedades cardiovasculares, el diagnóstico oportuno y la organización de la red de atención de estas enfermedades en el Sistema Único de Salud (Sistema Único de Saúde, SUS).

**Ethical aspects**

This research used public domain anonymized databases.

## Introduction

Cardiovascular diseases are the leading cause of death in Brazil and worldwide, with acute myocardial infarction standing out as a major contributor to morbidity and mortality and imposing a substantial burden on healthcare services^1^^,^^2^. In Brazil, mortality from diseases of the circulatory system remains a major public health concern, with a heterogeneous distribution across regions and population groups^3^.

Over recent decades, mortality rates from ischemic heart disease have declined, a trend attributed to improvements in prevention strategies, greater access to diagnosis, and advances in treatment^1^^,^^4^^-^^6^. However, this decline has not occurred uniformly across different regions and population groups. Differences in the organization of health services, access to timely care, and socioeconomic conditions contribute to the persistence of disparities in cardiovascular mortality^3^^,^^7^. Understanding these disparities is essential for planning and evaluating the actions implemented within the Brazilian Unified Health System (*Sistema Único de Saúde*, SUS).

Population aging is a key factor influencing the dynamics of acute myocardial infarction mortality in Brazil, with an increasing proportion of individuals in higher-risk age groups^8^^,^^9^. This process is likely to sustain a high absolute number of deaths despite declining age-standardized mortality rates, thereby increasing the demand for continuous care and for the organization of the healthcare network.

The COVID-19 pandemic introduced substantial changes in access to and use of healthcare services, with potential impacts on the management of cardiovascular diseases^10^^-^^13^. In Italy, between 12 and 19 March 2020, hospitalizations for acute myocardial infarction were 48% lower than during the corresponding week in 2019; in Northern California, weekly hospitalizations declined by up to 48% during the first weeks of the pandemic ^10^^,^^11^. In Brazil, the indirect effects of the pandemic on mortality from acute myocardial infarction have not yet been fully characterized, particularly in nationwide temporal analyses.

Analyzing mortality trends from acute myocardial infarction according to regional differences, sex, and age contributes to epidemiological surveillance and supports health planning. Studies using nationwide data enable monitoring of disease occurrence patterns, identifying disparities, and guiding interventions aimed at prevention, timely diagnosis, and the organization of the healthcare network for cardiovascular diseases.

The objective of this study was to analyze mortality from acute myocardial infarction in Brazil according to temporal trends, sociodemographic characteristics, and regional disparities from 2015 to 2024.

## Methods 

Study design

This was an ecological time series study using annual aggregated data to analyze mortality trends from acute myocardial infarction in Brazil from 2015 to 2024. 

Data source and case definition

Mortality data were obtained from the Mortality Information System (*Sistema de Informações sobre Mortalidade*, SIM), made available by the Department of Informatics of the Brazilian Unified Health System. Deaths with an underlying cause classified as acute myocardial infarction (ICD-10: I21), recorded in Brazil during the study period, were included.

Participants and stratifications

Population denominators were obtained from the intercensal population estimates produced by the Brazilian Institute of Geography and Statistics (*Instituto Brasileiro de Geografia e Estatística*, IBGE). Analyses were stratified by geographic region (North, Northeast, Southeast, South, and Central-West), sex (male and female), and age group (40-49, 50-59, 60-69, 70-79, and ≥80 years).

Study variables and data quality

Sociodemographic and death-related variables were analyzed, including age, sex, race/color, educational level (years of schooling), marital status, and place of occurrence. Data quality was assessed by calculating the proportion of records with missing or unknown information for the main variables, according to year of death.

Measures of occurrence

Absolute numbers of deaths and proportions were calculated, as well as unadjusted mortality rates per 100,000 inhabitants. Age-specific mortality rates were estimated for the predefined age groups. Age-standardized mortality rates were calculated using the direct method, with the world standard population and the Brazilian population as reference populations, the latter being used as a complementary analysis^14^. Additionally, age-standardized regional mortality rates were estimated using the Brazilian population as a common reference to evaluate the influence of differences in age structure on regional mortality patterns.

Statistical analysis

Temporal trends were assessed using log-linear regression, with the natural logarithm of the mortality rate as the dependent variable and calendar year as the independent variable. The annual percent change (APC) and corresponding 95% confidence intervals (95%CI) were estimated from the model slope coefficient. The APC, 95% confidence intervals, and p-values were estimated for age-standardized mortality rates, unadjusted mortality rates, geographic regions, sex, and age groups. Serial autocorrelation of the residuals was assessed using formal tests and graphical inspection. As a complementary analysis, temporal trends were reestimated using the Prais-Winsten method while maintaining the same log-linear model specification. To assess differences between sexes, mortality rate ratios were estimated using count regression models with population adjustment. Data were organized and analyzed using R software, version 4.5.2. The consolidated dataset used in the analysis is publicly available in a public repository^15^.

## Results

Between 2015 and 2024, a total of 938,997 deaths from acute myocardial infarction were recorded in Brazil. The annual number of deaths remained relatively stable throughout the study period, ranging from 90,465 in 2020 (9.6%) to 98,019 in 2022 (10.4%). Comparing 2015 (90,811 deaths) with 2024 (94,248 deaths), a modest increase of 3.8% was observed. The regional distribution showed the highest concentration of deaths in the Southeast (46.0%), followed by the Northeast (27.8%), South (13.5%), Central-West (6.9%), and North (5.8%).

Mortality exhibited a pronounced age gradient, with the highest proportions occurring among individuals aged 80 years or older (26.7%), 70-79 years (25.3%), and 60-69 years (23.8%), whereas individuals younger than 40 years accounted for less than 3.0% of deaths. A higher frequency of deaths was observed among males (59.3%). Regarding race/color, most deaths occurred among White (51.4%) and Brown (Brazilian mixed race) individuals (37.6%). With respect to educational level, the most frequent categories were 1-3 years (22.5%)and 4-7 years of schooling (22.9%). Deaths occurred most frequently in hospitals (49.2%), followed by deaths at home (34.8%) (Table 1).

Completeness was high for age, sex, and place of death, with less than 0.2% of records containing missing or unknown information. Greater incompleteness was observed for socioeconomic variables, particularly educational attainment.

**Table 1:** Characteristics of deaths from acute myocardial infarction. Brazil, 2015-2024 (n=938,997)

Variable	n (%)
Year	
2015	90,811 (9.7)
2016	94,148 (10.0)
2017	92,657 (9.9)
2018	93,272 (9.9)
2019	95,557 (10.2)
2020	90,465 (9.6)
2021	95,812 (10.2)
2022	98,019 (10.4)
2023	94,008 (10.0)
2024	94,248 (10.0)
Region	
North	54,740 (5.8)
Northeast	260,701 (27.8)
Southeast	432,273 (46.0)
South	126,515 (13.5)
Central-West	64,768 (6.9)
Age group (years)	
0-14	188 (<0.1)
15-19	960 (0.1)
20-29	5,564 (0.6)
30-39	19,201 (2.0)
40-49	60,623 (6.5)
50-59	139,037 (14.8)
60-69	223,939 (23.8)
70-79	237,692 (25.3)
80 or older	251,123 (26.7)
No information	670 (0.1)
Sex	
Male	556,761 (59.3)
Female	382,156 (40.7)
No information	80 (<0.1)
Race/color	
White	482,955 (51.4)
Black	75,640 (8.1)
Asian	5,531 (0.6)
Brown (Brazilian mixed race)	352,776 (37.6)
Indigenous	2,016 (0.2)
No information	20,079 (2.1)
Educational level (years of schooling)	
None	163,182 (17.4)
1-3	211,741 (22.5)
4-7	214,844 (22.9)
8-11	158,037 (16.8)
12 years or more	53,899 (5.7)
No information	137,294 (14.6)
Marital status	
Single	196,242 (20.9)
Married	360,770 (38.4)
Widowed	217,190 (23.1)
Legally separated	74,344 (7.9)
Other	34,143 (3.6)
No information	56,308 (6.0)
Place of death	
Hospital	461,792 (49.2)
Other healthcare facility	97,856 (10.4)
At home	326,805 (34.8)
Public road	17,310 (1.8)
Other	34,756 (3.7)
No information	478 (0.1)

The time series of the number of deaths showed moderate fluctuations throughout the study period, with an increase between 2015 and 2019, a decline in 2020, a subsequent increase through 2022, and stabilization in the most recent years (Figure 1).

Mortality rates showed persistent regional heterogeneity, with the highest levels observed in the Southeast, followed by the Northeast, and the lowest in the North. In three of the five regions, the highest mortality rates during the study period were observed in 2021 or 2022: the North, with 33.25 deaths per 100,000 inhabitants in 2021; the Southeast, with 51.20 per 100,000 inhabitants in 2022; and the Central-West, with 42.63 per 100,000 inhabitants in 2022. In subsequent years, mortality rates declined in the Southeast and Central-West, while fluctuations were observed in the North (Supplementary Table 1).

After age standardization using the Brazilian population as a common reference, differences in the regional pattern of mean mortality rates were observed. The highest mean age-standardized mortality rates were observed in the Northeast (120.97 per 100,000 inhabitants) and Central-West (116.14 per 100,000 inhabitants), followed by the Southeast (112.48 per 100,000 inhabitants), North (112.12 per 100,000 inhabitants), and South (96.58 per 100,000 inhabitants). These findings indicate that part of the differences observed in regional unadjusted mortality rates is related to differences in the age structure of regional populations.

Mortality rates were consistently higher among males throughout the study period. In 2015, the mortality rate among males was 53.96 per 100,000 population, compared with 36.16 among females. This difference widened over time, reaching an approximate rate ratio of 1.58 in 2024.

Age-specific mortality rates increased progressively with advancing age, with the highest rates observed in the oldest age groups. During the study period, a transient increase in mortality rates was observed, particularly among older adults, followed by a decline in the most recent years (Supplementary Table 2).

**Figure 1:**
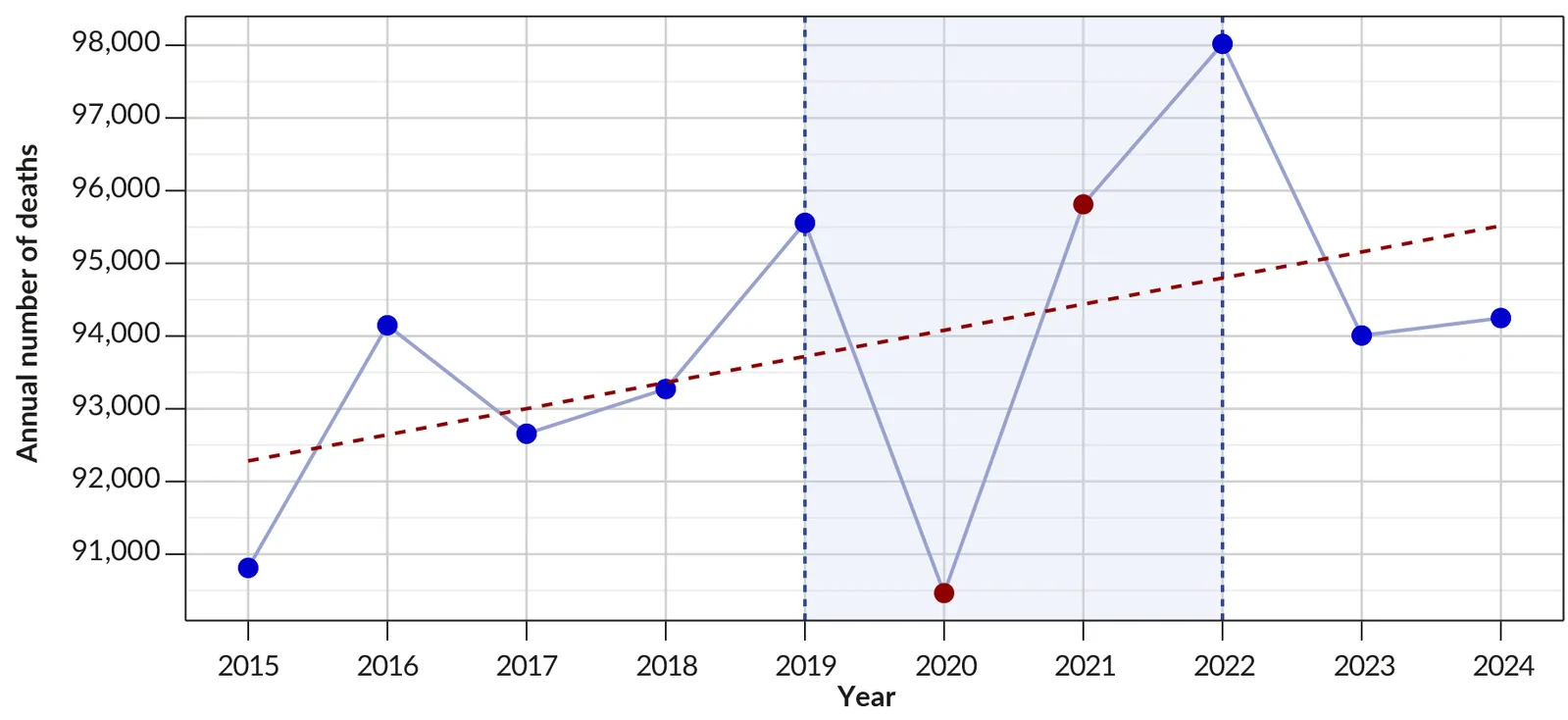
Annual number of deaths from acute myocardial infarction. Brazil, 2015-2024 (n=938,997)

^a^Gray line indicates annual number of deaths; ^b^Red dashed line indicates linear trend; ^c^Shaded area indicates pandemic period; ^d^Red dots indicate years 2020 and 2021.

The unadjusted mortality rate remained relatively stable throughout the study period. In contrast, age-standardized mortality rates showed a consistent downward trend using both the world standard population and the Brazilian standard population (Figure 2).

Temporal trends according to indicator, region, sex, and age group are presented in Table 2. A significant decline in age-standardized mortality rates was observed using both the WHO standard population (APC, −2.62%; 95%CI -3.20; −2.03) and the Brazilian standard population (APC, −2.63%; 95%CI −3.21; −2.04). The unadjusted mortality rate showed a stationary trend (APC, −0.16%; 95%CI −0.73; 0.41). Across regions, an increasing trend was observed in the North (APC, 2.11%; 95%CI 1.37; 2.85) and a decreasing trend in the South (APC, −1.73%; 95%CI −2.75; −0.70), whereas the Northeast, Southeast, and Central-West showed stationary trends. No significant trends were observed according to sex. In contrast, all age groups analyzed showed significant declines in mortality rates, with APC ranging from −1.83% among individuals aged 40-49 years to −2.95% among those aged 70-79 years.

**Table 2:** Temporal trends in mortality rates from acute myocardial infarction, according to indicator, region, sex, and age group. Brazil, 2015-2024 (n=938,997)

Indicator/category	APC^a^ (%)	95%CI^b^	p-value	Trend^c^
Age-standardized mortality rate				
WHO standard	-2.62	-3.20; -2.03	<0.001	Decreasing
Brazilian standard	-2.63	-3.21; -2.04	<0.001	Decreasing
Unadjusted rate				
Brazil	-0.16	-0.73; 0.41	0.531	Stationary
Region				
North	2.11	1.37; 2.85	<0.001	Increasing
Northeast	-0.10	-0.73; 0.54	0.733	Stationary
Southeast	-0.11	-0.78; 0.56	0.706	Stationary
South	-1.73	-2.75; -0.70	0.005	Decreasing
Central-West	0.88	-0.06; 1.83	0.062	Stationary
Sex				
Male	0.07	-0.45; 0.60	0.758	Stationary
Female	-0.49	-1.15; 0.18	0.130	Stationary
Age group				
40-49 years	-1.83	-2.75; -0.89	0.002	Decreasing
50-59 years	-2.46	-3.15; -1.77	<0.001	Decreasing
60-69 years	-2.55	-3.22; -1.88	<0.001	Decreasing
70-79 years	-2.95	-3.52; -2.39	<0.001	Decreasing
80 years or older	-2.65	-3.51; -1.77	<0.001	Decreasing

a APC: annual percent change; ^b^95% CI: 95% confidence interval; ^c^Trend estimated by log-linear regression.

**Figure 2:**
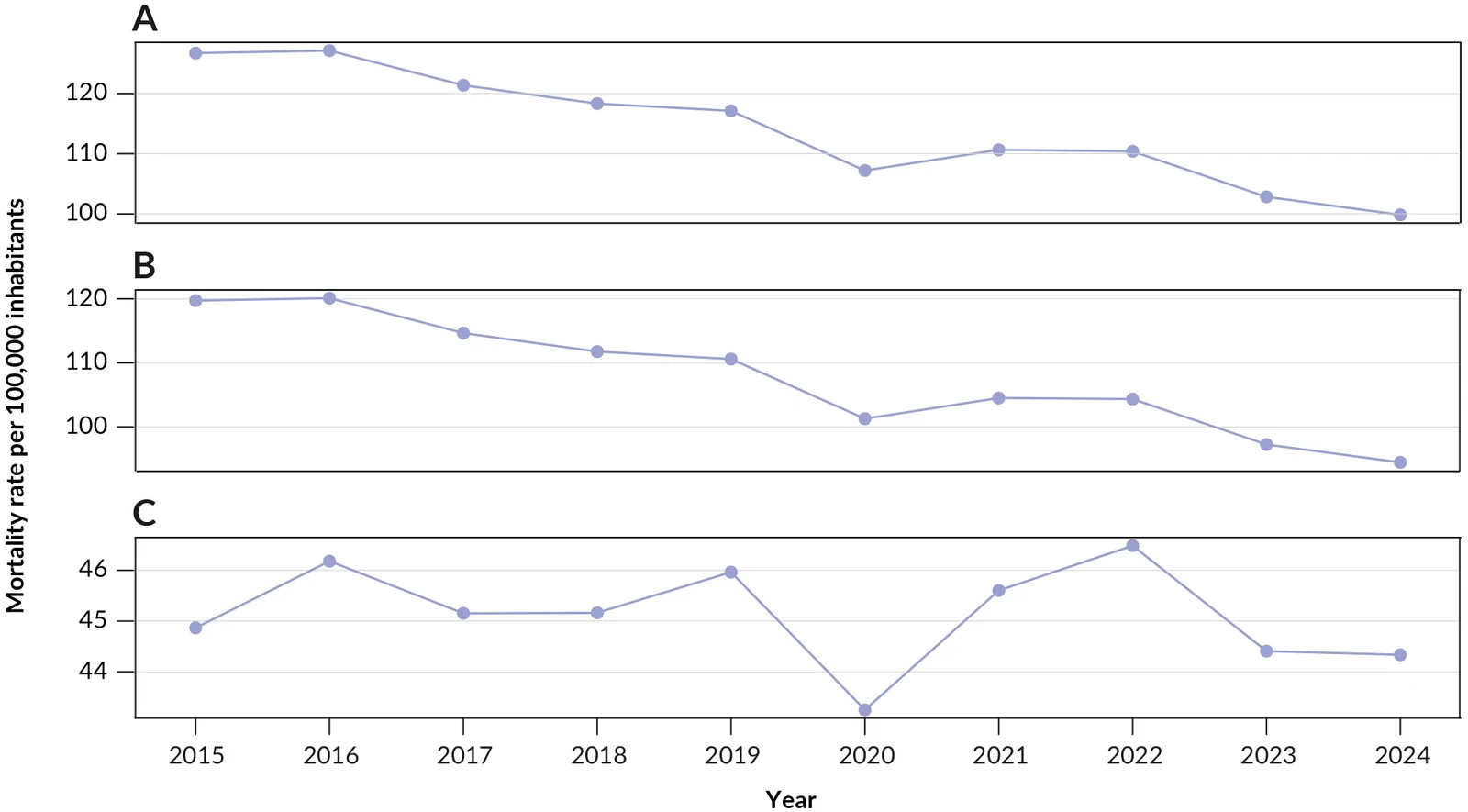
Unadjusted and age-standardized mortality rates from acute myocardial infarction. Brazil, 2015-2024 (n=938,997)

Legend: (A) Age-standardized mortality rate using the Brazilian population from the 2022 Population Census as the standard population; (B) Age-standardized mortality rate using the WHO world standard population (2000-2025); (C) Unadjusted mortality rate.

## Discussion

Deaths from acute myocardial infarction remained a substantial burden throughout the study period, with a consistent decline in age-standardized mortality rates coexisting with a stable absolute number of deaths. This pattern reflects the ongoing demographic transition, in which population aging sustains the magnitude of mortality despite advances in disease control^8^. Globally, between 1990 and 2023, the annual number of deaths from cardiovascular diseases increased from 13.1 million to 19.2 million, while prevalence rose from 311 million to 626 million cases. Concurrently, age-standardized rates showed a long-term decline, although population growth and aging contributed to an increase in the absolute burden of disease^6^^,^^9^. In the present study, deaths were concentrated among males and older age groups, and regional disparities persisted. These findings indicate that the observed gains were not distributed uniformly across the country.

The comparison between unadjusted and age-standardized mortality rates highlighted the important role of the age structure of the Brazilian population in the interpretation of mortality from acute myocardial infarction. Whereas the unadjusted mortality rate remained virtually stable throughout the study period, age-standardized rates showed a consistent decline, indicating that part of the persistence of the observed mortality levels may be attributed to population aging. In other words, after controlling for the effect of age, the risk of death from acute myocardial infarction in Brazil declined. This phenomenon illustrates the epidemiological relevance of age standardization in aging populations, as analyses based exclusively on unadjusted rates may obscure reductions in risk over time^6^^,^^8^^,^^9^.

Differences were also observed between age-standardized rates calculated using the world standard population and the Brazilian standard population. These differences reflect the distinct age distributions of the reference populations used. The Brazilian standard population assigns greater relative weight to older age groups, in which mortality from acute myocardial infarction is higher, resulting in slightly higher mortality estimates than those obtained using the world standard population. The use of the world standard population enhanced the international comparability of the findings, whereas the Brazilian standard population provided an interpretation more closely aligned with the national demographic context. The simultaneous use of both reference populations made it possible to evaluate the influence of age structure on mortality estimates and to broaden the epidemiological interpretation of the findings.

In Brazil, age-standardized mortality rates from cardiovascular diseases declined between 1990 and 2015, although with variations across states and regions^4^^,^^7^. Globally, age-standardized mortality rates from cardiovascular diseases also declined between 1990 and 2019, despite the persistence of a high absolute disease burden^1^^,^^6^. In the present study, regional heterogeneity was characterized by an increasing trend in the North (APC, 2.11%; 95%CI 1.37; 2.85), a decreasing trend in the South (APC, −1.73%; 95%CI −2.75; −0.70), and stationary trends in the Northeast, Southeast, and Central-West. These findings indicate territorial differences in the temporal evolution of mortality; however, the ecological design and the use of aggregated data do not allow identification of the underlying healthcare or socioeconomic mechanisms.

In Brazil, between 2019 and 2023, a total of 473,861 deaths from acute myocardial infarction were recorded, occurring more frequently among males, individuals aged 60 years or older, and residents of the Southeast region^16^. Between 2016 and 2022, deaths from acute ischemic heart diseases, classified under ICD-10 codes I21-I24, were concentrated among individuals aged 80 years or older and in the Southeast region; approximately 97% of these records corresponded to code I21^17^^,^^18^. In the present study, 59.3% of deaths occurred among males, 75.9% among individuals aged 60 years or older, and 46.0% in the Southeast region. A decline in age-standardized mortality rates was also observed despite the stability of the absolute number of deaths.

Across 57 European countries and neighboring regions, between 1990 and 2019, age-standardized cardiovascular mortality rates declined by 47% among males and 42% among females; despite this reduction, cardiovascular diseases remained the leading cause of death^5^. In the United States, between 2000 and 2023, a total of 933,024 deaths were recorded among adults aged 25 years or older in whom hypertension and acute myocardial infarction were listed concurrently. The age-adjusted mortality rate declined from 19.84 to 16.26 per 100,000 population but remained higher among males, non-Hispanic Black individuals, and residents of the South and rural areas^19^. At a tertiary hospital in Karachi, Pakistan, 14,601 hospitalizations for acute myocardial infarction between 1988 and 2018 were analyzed. Comparing the periods 1988-1997 and 2008-2018, the median age increased from 58 to 63 years, the prevalence of hypertension rose from 44% to 71%, and the prevalence of diabetes increased from 38% to 53%. In contrast, in-hospital mortality declined from 14% to 9%^20^.

In the present study, the annual number of deaths remained relatively stable, with a 3.8% increase between 2015 and 2024, whereas age-standardized mortality rates declined. Furthermore, 75.9% of deaths occurred among individuals aged 60 years or older. Taken together, these findings are consistent with the contribution of population aging to the persistence of the high absolute burden of mortality from acute myocardial infarction and reinforce the need for strategies aimed at healthy aging, multimorbidity management, and continuity of care^8^^,^^9^.

In the present study, age-standardized regional mortality rates using the Brazilian standard population ranged from 96.58 per 100,000 inhabitants in the South to 120.97 per 100,000 inhabitants in the Northeast. The persistence of this variation after age standardization indicates that regional differences are not explained exclusively by differences in age structure. In a systematic review and meta-analysis of 37 international studies published through January 2024, including 21.1 million patients with acute myocardial infarction, follow-up mortality was higher among residents of rural areas than among residents of urban areas (15.5% versus 13.4%; OR 1.18; 95%CI 1.13; 1.24)^21^. In the United States, race/color, place of residence, insurance coverage, income or poverty, and educational attainment were associated with mortality or readmission following acute myocardial infarction^22^. In contrast, women and racial minority groups experienced poorer outcomes and differences in disease management^23^. Across 40 cohorts and registries from the United States, the United Kingdom, and Canada, comprising 14.2 million patients with acute coronary syndrome, mortality following ST-segment elevation myocardial infarction was higher among Black individuals than among White individuals; among Asian individuals, higher mortality was identified in analyses conducted in the United States^24^.

The predominance of mortality among males is consistent with the literature and may be related to greater exposure to risk factors, lower use of preventive healthcare services, and behavioral differences^2^. However, the analysis of sex-related disparities should also consider differences in clinical presentation and access to care among women, which may influence health outcomes. In addition to behavioral factors, differences in diagnosis, clinical presentation, and access to treatment have been identified as possible determinants of the disparities observed between men and women following acute coronary events^23^. Prevention strategies should therefore take these differences into account by promoting interventions tailored to different population profiles.

In the present study, fluctuations in the number of deaths between 2020 and 2022 coincided with the COVID-19 pandemic, although the study design does not allow these changes to be causally attributed to COVID-19. In Italy, during one week of the initial phase of the pandemic in 2020, hospitalizations for acute myocardial infarction were 48% lower than during the corresponding week in 2019; in Northern California, weekly hospitalization rates declined by up to 48% during the first weeks of the pandemic^10^^,^^11^. Across 32 studies conducted in different countries, 19,140 patients with ST-segment elevation myocardial infarction underwent primary percutaneous coronary intervention during the pandemic, compared with 68,662 before the pandemic; door-to-balloon time increased by a mean of 8.10 minutes (95%CI 3.90; 12.30), and in-hospital mortality was higher (OR 1.27; 95%CI 1.09; 1.49)^12^^,^^13^. In a meta-analysis of 26 studies conducted in different countries, including 1,175,537 patients with COVID-19, myocardial infarction was associated with higher mortality (pooled effect 1.20; 95%CI 1.09; 1.32)^25^. In the United States, in 2020, 5,818 of 555,540 hospitalizations for myocardial infarction involved concomitant COVID-19; these cases were associated with lower use of coronary angiography, a higher frequency of cardiac complications, and higher in-hospital mortality^26^. These findings provide clinical and healthcare-related plausibility for interpreting the fluctuations observed in the present time series, although they do not support the establishment of a causal relationship.

The findings of this study indicate that reducing mortality from acute myocardial infarction requires integrated actions across different levels of healthcare. Across 18 randomized and cluster-randomized trials published between 2003 and 2024 and conducted in 13 countries, involving 438,285 individuals with cardiovascular diseases receiving primary or hospital care, quality improvement interventions increased the prescription of lipid-lowering medications (OR 1.46; 95%CI 1.08; 1.99) and reduced major cardiovascular events (OR 0.84; 95%CI 0.71; 0.98) and all-cause mortality (OR 0.87; 95%CI 0.77; 0.98)^27^. In a meta-analysis of 14 randomized clinical trials, predominantly conducted in China and including 1,463 patients with coronary artery disease undergoing percutaneous coronary intervention, structured continuous nursing care interventions after hospital discharge improved quality of life, cardiac function, and treatment adherence, while reducing adverse cardiovascular events (OR 0.18; 95%CI 0.09; 0.36)^28^. Reducing regional disparities should be considered a priority in the formulation of public policies aimed at building a healthcare network capable of responding to the high burden of cardiovascular diseases in Brazil^29^^,^^30^.

This study has limitations inherent to the use of secondary data, including possible misclassification and incomplete information, particularly for socioeconomic variables. Although data quality was high for essential variables, the presence of missing or unknown data may have introduced information bias. Additionally, the analysis based on aggregated data limits individual-level inference, restricting the assessment of clinical and contextual factors associated with mortality.

In summary, a high burden of mortality from acute myocardial infarction was observed throughout the study period, with predominance among older adults, higher occurrence among males, and persistent regional disparities. The decline in age-standardized mortality rates indicates a reduction in adjusted mortality despite the stability of the absolute number of deaths. These findings reinforce the need to strengthen prevention strategies, timely diagnosis, and the organization of the healthcare network for cardiovascular diseases within the Brazilian Unified Health System, with a focus on reducing disparities and preparing the health system for the growing impact of population aging on the cardiovascular disease burden in the coming decades^8^^,^^9^.

## Data Availability

The consolidated dataset used in the analysis is available in a public repository: https://doi.org/10.5281/zenodo.19488974^15^.
