# Supplementary material for: Temporal trends and regional disparities in mortality from acute myocardial infarction in Brazil: a time series study, 2015-2024
Source: Epidemiol Serv Saude. 2026 Sep 7;35:e20260533. doi: 10.1590/S2237-96222026v35e20260533.en (PMC13617566; doi:10.1590/S2237-96222026v35e20260533.en)
Supplement: Supplementary file 1 [file 2237-9622-ress-35-e20260533-sup-pt.pdf]

Tabelas Suplementares

Tabela Suplementar 1. Taxas de mortalidade por infarto agudo do miocárdio (por 100.000 habitantes) segundo região geográfica e ano. Brasil, 2015–2024

Ano	Norte	Nordeste	Sudeste	Sul	Centro-Oeste
2015	26,97	45,70	48,92	45,88	37,47
2016	27,53	46,27	50,95	46,78	39,26
2017	29,35	47,24	49,17	42,23	38,87
2018	29,65	47,00	49,03	42,72	39,49
2019	30,19	48,09	50,69	41,71	38,47
2020	30,66	44,33	47,48	39,08	38,54
2021	33,25	45,22	50,42	41,44	42,59
2022	31,53	47,12	51,20	42,84	42,63
2023	31,86	46,25	48,65	38,20	41,15
2024	32,58	45,98	48,47	39,36	39,31

Nota: Taxas brutas de mortalidade calculadas com base no número anual de óbitos por infarto agudo do miocárdio (CID-10: I21) e nas estimativas populacionais do Instituto Brasileiro de Geografia e Estatística (IBGE).

Tabela Suplementar 2. Taxas específicas de mortalidade por infarto agudo do miocárdio (por 100.000 habitantes) segundo faixa etária e ano. Brasil, 2015–2024.

Ano	40–49 anos	50–59 anos	60–69 anos	70–79 anos	80 anos ou mais
2015	23,32	66,19	150,69	305,92	668,23
2016	23,26	66,66	152,78	303,42	671,19
2017	22,05	62,87	146,78	289,41	643,29
2018	21,31	61,33	144,16	283,56	620,16
2019	20,56	60,86	142,18	277,98	625,16
2020	19,40	55,04	127,93	251,72	588,92
2021	19,90	56,39	131,51	260,37	612,11
2022	20,85	57,49	132,77	256,26	598,00
2023	19,91	54,85	125,38	240,82	533,44
2024	19,94	53,30	120,71	235,16	514,56

Nota: Taxas específicas de mortalidade calculadas para cada faixa etária utilizando o número anual de óbitos por infarto agudo do miocárdio (CID-10: I21) e as respectivas estimativas populacionais do Instituto Brasileiro de Geografia e Estatística (IBGE).
