# Supplementary material for: Temporal trends and regional disparities in mortality from acute myocardial infarction in Brazil: a time series study, 2015-2024
Source: Epidemiol Serv Saude. 2026 Sep 7;35:e20260533. doi: 10.1590/S2237-96222026v35e20260533.en (PMC13617566; doi:10.1590/S2237-96222026v35e20260533.en)
Supplement: Supplementary file 2 [file 2237-9622-ress-35-e20260533-sup.pdf]

Supplementary Tables

Supplementary Table 1. Mortality rates from acute myocardial infarction (per 100,000 inhabitants), by geographic region and year. Brazil, 2015–2024

Year	North	Northeast	Southeast	South	Central-West
2015	26.97	45.70	48.92	45.88	37.47
2016	27.53	46.27	50.95	46.78	39.26
2017	29.35	47.24	49.17	42.23	38.87
2018	29.65	47.00	49.03	42.72	39.49
2019	30.19	48.09	50.69	41.71	38.47
2020	30.66	44.33	47.48	39.08	38.54
2021	33.25	45.22	50.42	41.44	42.59
2022	31.53	47.12	51.20	42.84	42.63
2023	31.86	46.25	48.65	38.20	41.15
2024	32.58	45.98	48.47	39.36	39.31

Note: Unadjusted mortality rates were calculated based on the annual number of deaths from acute myocardial infarction (ICD-10: I21) and the corresponding population estimates from the Brazilian Institute of Geography and Statistics (*Instituto Brasileiro de Geografia e Estatística*, IBGE).

Supplementary Table 2. Specific mortality rates from acute myocardial infarction (per 100,000 inhabitants) according to age group and year. Brazil, 2015–2024

Year	40–49 years	50–59 years	60–69 years	70–79 years	80 years or older
2015	23.32	66.19	150.69	305.92	668.23
2016	23.26	66.66	152.78	303.42	671.19
2017	22.05	62.87	146.78	289.41	643.29
2018	21.31	61.33	144.16	283.56	620.16
2019	20.56	60.86	142.18	277.98	625.16
2020	19.40	55.04	127.93	251.72	588.92
2021	19.90	56.39	131.51	260.37	612.11
2022	20.85	57.49	132.77	256.26	598.00
2023	19.91	54.85	125.38	240.82	533.44
2024	19.94	53.30	120.71	235.16	514.56

Note: Age-specific mortality rates were calculated for each age group using the annual number of deaths from acute myocardial infarction (ICD-10: I21) and the corresponding population estimates from the Brazilian Institute of Geography and Statistics (*Instituto Brasileiro de Geografia e Estatística*, IBGE).
