# Supplementary material for: Temporal trends and regional disparities in mortality from acute myocardial infarction in Brazil: a time series study, 2015-2024
Source: Epidemiol Serv Saude. 2026 Sep 7;35:e20260533. doi: 10.1590/S2237-96222026v35e20260533.en (PMC13617566; doi:10.1590/S2237-96222026v35e20260533.en)
Supplement: Translation

# Translation: Tendências temporais e disparidades regionais da mortalidade por infarto agudo do miocárdio no Brasil: estudo de série temporal, 2015-2024

**DOI:** 10.1590/S2237-96222026v35e20260533.pt

**Authors:** Geovanna Aires Silva, Carlos Henrique Santos, Jacqueline Andréia Bernardes Leão-Cordeiro, Antonio Marcio Teodoro Cordeiro Silva

**Affiliations:** 1 Pontifícia Universidade Católica de Goiás, Medicina, Goiânia, GO, Brasil Pontifícia Universidade Católica de Goiás Medicina Goiânia GO Brasil; 2 Universidade Federal de Goiás, Enfermagem, Goiânia, GO, Brasil Universidade Federal de Goiás Enfermagem Goiânia GO Brasil

**Keywords:** Doenças Cardiovasculares, Envelhecimento, Disparidades nos Níveis de Saúde, Sistema Único de Saúde, Estudos de Séries Temporais

## Abstract

**Objetivo:**

Analisar a mortalidade por infarto agudo do miocárdio no Brasil, segundo tendências temporais, características sociodemográficas e desigualdades regionais, no período de 2015 a 2024.

**Métodos:**

Estudo de série temporal com dados agregados anuais do Sistema de Informações sobre Mortalidade. Foram calculadas taxas de mortalidade brutas, taxas padronizadas por idade e razões de taxas segundo sexo. As tendências temporais foram avaliadas por regressão log-linear, com estimativa da variação percentual anual (*Annual Percent Change*, APC), e reestimadas pelo método de Prais-Winsten como análise complementar.

**Resultados:**

Foram registrados 938.997 óbitos no período. O número absoluto de óbitos apresentou estabilidade, com aumento de 3,8% entre 2015 e 2024. As taxas padronizadas por idade diminuíram ao longo da série (variação percentual anual -2,6%; p-valor<0,001). A mortalidade foi maior entre homens, com razão média de taxas de 1,53 no período analisado, alcançando 1,58 em 2024, e concentrou-se em indivíduos idosos. As regiões Sudeste e Nordeste apresentaram as maiores taxas brutas de mortalidade.

**Conclusão:**

Observou-se redução das taxas de mortalidade padronizadas por idade ao longo do período analisado, embora tenha persistido elevada carga de óbitos associada ao envelhecimento populacional e a desigualdades regionais. Esses achados reforçam a necessidade de fortalecer ações de prevenção cardiovascular, diagnóstico oportuno e organização da rede de atenção às doenças cardiovasculares no Sistema Único de Saúde.

**Aspectos éticos**

Esta pesquisa utilizou bancos de dados de domínio público e anonimizados.

## Introdução

As doenças cardiovasculares são a principal causa de morte no Brasil e no mundo, com destaque para o infarto agudo do miocárdio, responsável por elevada carga de morbimortalidade e impacto nos serviços de saúde^1^^,^^2^. No país, a mortalidade por doenças do aparelho circulatório mantém-se como importante problema de saúde pública, com distribuição heterogênea entre regiões e grupos populacionais^3^.

Nas últimas décadas, as taxas de mortalidade por doenças isquêmicas do coração apresentaram redução, atribuída ao aprimoramento das estratégias de prevenção, ao maior acesso ao diagnóstico e aos avanços terapêuticos^1^^,^^4^^-^^6^. Contudo, esse declínio não ocorreu de forma homogênea entre as diferentes regiões e grupos populacionais. Diferenças na organização dos serviços de saúde, no acesso ao cuidado oportuno e nas condições socioeconômicas contribuem para que desigualdades na mortalidade cardiovascular persistam^3^^,^^7^. Compreender essas disparidades é fundamental para planejar e avaliar as ações desenvolvidas no Sistema Único de Saúde.

O envelhecimento populacional constitui fator central na dinâmica da mortalidade por infarto agudo do miocárdio no Brasil, com aumento da proporção de indivíduos em faixas etárias de maior risco^8^^,^^9^. Esse processo tende a manter elevada a carga absoluta de óbitos, mesmo diante da redução das taxas padronizadas, o que amplia a demanda por cuidados contínuos e por organização da rede assistencial.

A pandemia de covid-19 introduziu mudanças relevantes no acesso e na utilização dos serviços de saúde, com impacto potencial sobre o manejo das doenças cardiovasculares^10^^-^^13^. Na Itália, entre 12 e 19 de março de 2020, as internações por infarto agudo do miocárdio foram 48% menores que na semana correspondente de 2019; no norte da Califórnia, as internações semanais diminuíram em até 48% nas primeiras semanas da pandemia^10^^,^^11^. No Brasil, os efeitos indiretos da pandemia sobre a mortalidade por infarto agudo do miocárdio ainda não estão completamente caracterizados, especialmente em análises nacionais com abordagem temporal.

A análise das tendências de mortalidade por infarto agudo do miocárdio, considerando diferenças regionais, sexo e idade, contribui para a vigilância epidemiológica e subsidia o planejamento das ações em saúde. Estudos com dados nacionais permitem monitorar padrões de ocorrência, identificar desigualdades e orientar intervenções voltadas à prevenção, ao diagnóstico oportuno e à organização da rede de atenção às doenças cardiovasculares.

O objetivo deste estudo foi analisar a mortalidade por infarto agudo do miocárdio no Brasil, segundo tendências temporais, características sociodemográficas e desigualdades regionais, no período de 2015 a 2024.

## Métodos

Desenho do estudo

Trata-se de um estudo ecológico de série temporal, utilizando dados agregados anuais para análise das tendências da mortalidade por infarto agudo do miocárdio no Brasil, no período de 2015 a 2024.

Fonte de dados e definição de caso

Os dados de mortalidade foram obtidos do Sistema de Informações sobre Mortalidade, disponibilizado pelo Departamento de Informática do Sistema Único de Saúde. Foram incluídos os óbitos cuja causa básica foi classificada como infarto agudo do miocárdio (CID-10: I21), registrados no Brasil no período do estudo.

População e estratificações

As populações utilizadas como denominadores foram obtidas das estimativas intercensitárias do Instituto Brasileiro de Geografia e Estatística. As análises foram estratificadas por região geográfica (Norte, Nordeste, Sudeste, Sul e Centro-Oeste); sexo (masculino e feminino); e faixas etárias (40-49, 50-59, 60-69, 70-79 e 80 anos ou mais).

Variáveis do estudo e qualidade da informação

Foram analisadas variáveis sociodemográficas e relacionadas ao óbito, incluindo idade, sexo, raça/cor, escolaridade (em anos de estudo), estado civil e local de ocorrência. A qualidade da informação foi avaliada por meio da proporção de registros com informação ignorada ou ausente para as principais variáveis, segundo ano do óbito.

Medidas de ocorrência

Foram calculados números absolutos de óbitos e proporções, além de taxas brutas de mortalidade por 100 mil habitantes. As taxas específicas por idade foram estimadas para os grupos etários definidos. Foram calculadas taxas padronizadas por idade pelo método direto, utilizando como referência a população padrão mundial e a população brasileira, sendo esta última empregada como análise complementar^14^. Adicionalmente, foram estimadas taxas regionais padronizadas por idade utilizando a população brasileira como referência comum, com o objetivo de avaliar a influência das diferenças na estrutura etária sobre os padrões regionais de mortalidade.

Análise estatística

A tendência temporal foi avaliada por regressão log-linear, utilizando como variável dependente o logaritmo natural da taxa de mortalidade e como variável independente o ano. A partir do coeficiente angular do modelo, foi estimada a variação percentual anual (*Annual Percent Change*, APC), com respectivos intervalos de confiança de 95% (IC95%). Foram estimados APC, intervalos de confiança de 95% e p-valores para as taxas padronizadas por idade, taxa bruta, regiões geográficas, sexo e faixas etárias. A presença de autocorrelação serial dos resíduos foi investigada por testes formais e inspeção gráfica. Como análise complementar, a tendência foi reestimada pelo método de Prais-Winsten, mantendo-se a mesma estrutura log-linear. Para avaliar diferenças entre os sexos, foram estimadas razões de taxas de mortalidade utilizando modelos de regressão para contagem com ajuste pela população. Os dados foram organizados e analisados com uso do software R, versão 4.5.2. O banco de dados consolidado utilizado na análise está disponível em repositório público^15^.

## Resultados

Entre 2015 e 2024, foram registrados 938.997 óbitos por infarto agudo do miocárdio no Brasil. O número anual de óbitos apresentou relativa estabilidade ao longo da série, variando de 90.465 em 2020 (9,6%) a 98.019 em 2022 (10,4%). Comparando-se 2015 (90.811 óbitos) e 2024 (94.248), observou-se incremento discreto de 3,8%. A distribuição regional evidenciou maior concentração no Sudeste (46,0%), seguido do Nordeste (27,8%), Sul (13,5%), Centro-Oeste (6,9%) e Norte (5,8%).

A mortalidade apresentou forte gradiente etário, com predomínio nas faixas de 80 anos ou mais (26,7%), 70-79 anos (25,3%) e 60-69 anos (23,8%), enquanto indivíduos com menos de 40 anos representaram menos de 3,0% dos óbitos. Observou-se maior frequência no sexo masculino (59,3%). Quanto à raça/cor, predominaram indivíduos brancos (51,4%) e pardos (37,6%). Em relação à escolaridade, destacaram-se as categorias de 1-3 anos (22,5%) e 4-7 anos (22,9%). Os óbitos ocorreram mais frequentemente em ambiente hospitalar (49,2%), seguidos pelo domicílio (34,8%) (Tabela 1).

A completude das variáveis foi elevada para idade, sexo e local do óbito, com proporção de registros ignorados inferior a 0,2%. Para variáveis socioeconômicas, observou-se maior incompletude, especialmente para escolaridade.

**Tabela 1:** Características dos óbitos por infarto agudo do miocárdio. Brasil, 2015-2024 (n=938.997)

Variável	n (%)
Ano	
2015	90.811 (9,7)
2016	94.148 (10,0)
2017	92.657 (9,9)
2018	93.272 (9,9)
2019	95.557 (10,2)
2020	90.465 (9,6)
2021	95.812 (10,2)
2022	98.019 (10,4)
2023	94.008 (10,0)
2024	94.248 (10,0)
Região	
Norte	54.740 (5,8)
Nordeste	260.701 (27,8)
Sudeste	432.273 (46,0)
Sul	126.515 (13,5)
Centro-Oeste	64.768 (6,9)
Faixa etária (anos)	
0-14	188 (<0,1)
15-19	960 (0,1)
20-29	5.564 (0,6)
30-39	19.201 (2,0)
40-49	60.623 (6,5)
50-59	139.037 (14,8)
60-69	223.939 (23,8)
70-79	237.692 (25,3)
80 ou mais	251.123 (26,7)
Sem informação	670 (0,1)
>Sexo	
Masculino	556.761 (59,3)
Feminino	382.156 (40,7)
Sem informação	80 (<0,1)
Raça/cor da pele	
Branca	482.955 (51,4)
Preta	75.640 (8,1)
Amarela	5.531 (0,6)
Parda	352.776 (37,6)
Indígena	2.016 (0,2)
Sem informação	20.079 (2,1)
Escolaridade (anos)	
Nenhuma	163.182 (17,4)
1-3	211.741 (22,5)
4-7	214.844 (22,9)
8-11	158.037 (16,8)
12 ou mais	53.899 (5,7)
Sem informação	137.294 (14,6)
Estado civil	
Solteiro	196.242 (20,9)
Casado	360.770 (38,4)
Viúvo	217.190 (23,1)
Separado judicialmente	74.344 (7,9)
Outro	34.143 (3,6)
Sem informação	56.308 (6,0)
Local do óbito	
Hospital	461.792 (49,2)
Outro estabelecimento de saúde	97.856 (10,4)
Domicílio	326.805 (34,8)
Via pública	17.310 (1,8)
Outros	34.756 (3,7)
Sem informação	478 (0,1)

A série temporal do número de óbitos apresentou flutuações moderadas ao longo do período, com aumento entre 2015 e 2019, redução em 2020 e elevação até 2022, seguida de estabilização nos anos mais recentes (Figura 1).

As taxas de mortalidade apresentaram heterogeneidade regional persistente, com maiores níveis no Sudeste, seguido do Nordeste, e menores no Norte. Em três das cinco regiões, os maiores valores da série foram observados em 2021 ou 2022: Norte, com 33,25 óbitos por 100 mil habitantes em 2021; Sudeste, com 51,20 por 100 mil habitantes em 2022; e Centro-Oeste, com 42,63 por 100 mil habitantes em 2022. Nos anos subsequentes, observaram-se redução no Sudeste e no Centro-Oeste e oscilações no Norte (Tabela Suplementar 1).

Após padronização por idade utilizando a população brasileira como referência comum, observaram-se diferenças no padrão regional das taxas médias de mortalidade. As maiores taxas padronizadas médias foram observadas no Nordeste (120,97 por 100 mil habitantes) e no Centro-Oeste (116,14 por 100 mil habitantes), seguidas do Sudeste (112,48 por 100 mil habitantes), Norte (112,12 por 100 mil habitantes) e Sul (96,58 por 100 mil habitantes). Esses resultados indicam que parte das diferenças observadas nas taxas regionais brutas está relacionada à estrutura etária das populações regionais.

As taxas de mortalidade foram consistentemente maiores entre homens ao longo de todo o período. Em 2015, a taxa masculina foi de 53,96 por 100 mil habitantes, comparada a 36,16 entre mulheres, diferença que se ampliou ao longo da série, atingindo razão aproximada de 1,58 em 2024.

As taxas específicas por idade aumentaram progressivamente com o envelhecimento, sendo mais elevadas nas faixas etárias mais avançadas. Durante o período, observou-se elevação transitória das taxas, especialmente entre idosos, seguida de redução nos anos mais recentes (Tabela Suplementar 2).

**Figura 1:** Número anual de óbitos por infarto agudo do miocárdio. Brasil, 2015-2024 (n=938.997)

A taxa bruta apresentou relativa estabilidade ao longo do período, enquanto as taxas padronizadas por idade mostraram tendência de redução consistente ao longo da série, tanto pela população padrão mundial quanto pela população brasileira (Figura 2).

As tendências temporais segundo indicador, região, sexo e faixa etária são apresentadas na Tabela 2. 

Observou-se redução significativa das taxas padronizadas por idade, tanto pelo padrão OMS (APC -2,62%; IC95% -3,20; -2,03) quanto pelo padrão brasileiro (APC -2,63%; IC95% -3,21; -2,04). A taxa bruta apresentou tendência estacionária (APC -0,16%; IC95% -0,73; 0,41). Entre as regiões, verificou-se tendência crescente no Norte (APC 2,11%; IC95% 1,37; 2,85) e decrescente no Sul (APC -1,73%; IC95% -2,75; -0,70), enquanto Nordeste, Sudeste e Centro-Oeste apresentaram tendências estacionárias. Não foram observadas tendências significativas segundo sexo. Em contrapartida, todas as faixas etárias avaliadas apresentaram redução significativa das taxas de mortalidade, com APC variando de -1,83% entre indivíduos de 40-49 anos a -2,95% entre aqueles de 70-79 anos.

**Tabela 2:** Tendência temporal das taxas de mortalidade por infarto agudo do miocárdio, segundo indicador, região, sexo e faixa etária. Brasil, 2015-2024 (n=938.997)

Indicador/categoria	APC^a^ (%)	IC95%^b^	p-valor	Tendência^c^
Taxa padronizada por idade				
Padrão OMS	-2,62	-3,20; -2,03	<0,001	Decrescente
Padrão Brasil	-2,63	-3,21; -2,04	<0,001	Decrescente
Taxa bruta				
Brasil	-0,16	-0,73; 0,41	0,531	Estacionária
Região				
Norte	2,11	1,37; 2,85	<0,001	Crescente
Nordeste	-0,10	-0,73; 0,54	0,733	Estacionária
Sudeste	-0,11	-0,78; 0,56	0,706	Estacionária
Sul	-1,73	-2,75; -0,70	0,005	Decrescente
Centro-Oeste	0,88	-0,06; 1,83	0,062	Estacionária
Sexo				
Masculino	0,07	-0,45; 0,60	0,758	Estacionária
Feminino	-0,49	-1,15; 0,18	0,130	Estacionária
Faixa etária				
40-49 anos	-1,83	-2,75; -0,89	0,002	Decrescente
50-59 anos	-2,46	-3,15; -1,77	<0,001	Decrescente
60-69 anos	-2,55	-3,22; -1,88	<0,001	Decrescente
70-79 anos	-2,95	-3,52; -2,39	<0,001	Decrescente
80 anos ou mais	-2,65	-3,51; -1,77	<0,001	Decrescente

a APC: Annual Percent Change; ^b^IC95%: intervalo de confiança de 95%; ^c^Tendência estimada por regressão log-linear.

**Figura 2:** Taxa bruta e taxas padronizadas por idade da mortalidade por infarto agudo do miocárdio. Brasil, 2015-2024 (n=938.997)

## Discussão 

Os óbitos por infarto agudo do miocárdio mantiveram elevada carga ao longo do período analisado, com redução consistente das taxas padronizadas por idade, coexistindo com estabilidade do número absoluto de óbitos. Esse padrão reflete a transição demográfica em curso, na qual o envelhecimento populacional sustenta a magnitude da mortalidade, mesmo diante de avanços no controle da doença^8^. Em âmbito global, entre 1990 e 2023, o número anual de mortes por doenças cardiovasculares aumentou de 13,1 milhões para 19,2 milhões, enquanto a prevalência passou de 311 milhões para 626 milhões de casos. Paralelamente, as taxas padronizadas por idade apresentaram declínio de longo prazo, mas o crescimento e o envelhecimento populacionais contribuíram para o aumento da carga absoluta da doença^6^^,^^9^. Neste estudo, os óbitos concentraram-se entre homens e indivíduos de faixas etárias mais avançadas, e persistiram desigualdades regionais. Esses resultados indicam que os ganhos observados não ocorreram de forma homogênea no território nacional.

A comparação entre as taxas brutas e as taxas padronizadas evidenciou o importante papel da estrutura etária da população brasileira na interpretação da mortalidade por infarto agudo do miocárdio. Enquanto a taxa bruta apresentou tendência praticamente estável ao longo do período, as taxas padronizadas mostraram redução consistente, indicando que parte da manutenção dos níveis observados de mortalidade pode ser atribuída ao envelhecimento populacional. Em outras palavras, após o controle do efeito da idade, observa-se diminuição do risco de morte por infarto agudo do miocárdio no país. Esse fenômeno ilustra a relevância epidemiológica da padronização por idade em populações em processo de envelhecimento, pois a análise exclusiva de taxas brutas pode mascarar a redução do risco ao longo do tempo^6^^,^^8^^,^^9^.

Também foram observadas diferenças entre as taxas padronizadas calculadas com base na população padrão mundial e na população padrão brasileira. Essas diferenças refletem as distintas composições etárias dos padrões de referência utilizados. A população padrão brasileira atribui maior peso relativo às faixas etárias mais avançadas, nas quais a mortalidade por infarto agudo do miocárdio é mais elevada, resultando em estimativas de mortalidade ligeiramente superiores às obtidas com o padrão mundial. A utilização da população padrão mundial favoreceu a comparabilidade internacional dos resultados, enquanto a população padrão brasileira permitiu interpretação mais aderente ao contexto demográfico nacional. O emprego simultâneo desses referenciais possibilitou avaliar a influência da estrutura etária sobre as estimativas de mortalidade e ampliar a interpretação epidemiológica dos achados.

No Brasil, as taxas padronizadas de mortalidade por doenças cardiovasculares diminuíram entre 1990 e 2015, embora com variações entre estados e regiões^4^^,^^7^. Em âmbito global, entre 1990 e 2019, também foi registrada redução das taxas ajustadas por idade para doenças cardiovasculares, apesar da manutenção de elevada carga absoluta^1^^,^^6^. No presente estudo, a heterogeneidade regional foi expressa por tendência crescente no Norte (APC 2,11%; IC95% 1,37; 2,85), decrescente no Sul (APC -1,73%; IC95% -2,75; -0,70) e estacionária no Nordeste, Sudeste e Centro-Oeste. Esses resultados indicam diferenças territoriais na evolução temporal da mortalidade; contudo, o delineamento ecológico e o uso de dados agregados não permitem identificar os mecanismos assistenciais ou socioeconômicos subjacentes.

No Brasil, entre 2019 e 2023, foram registrados 473.861 óbitos por infarto agudo do miocárdio, com maior frequência entre homens, pessoas com 60 anos ou mais e residentes no Sudeste^16^. Entre 2016 e 2022, os óbitos por doenças isquêmicas agudas do coração, classificados nos códigos I21-I24, concentraram-se em pessoas com 80 anos ou mais e na região Sudeste; aproximadamente 97% dos registros corresponderam ao código I21^17^^,^^18^. No presente estudo, 59,3% dos óbitos ocorreram em homens, 75,9% em pessoas com 60 anos ou mais e 46,0% no Sudeste. Também se verificou redução das taxas padronizadas por idade, apesar da estabilidade do número absoluto de óbitos.

Em 57 países europeus e de regiões adjacentes, entre 1990 e 2019, as taxas padronizadas de mortalidade cardiovascular diminuíram 47% entre homens e 42% entre mulheres; apesar dessa redução, as doenças cardiovasculares permaneceram como a principal causa de morte^5^. Nos Estados Unidos, entre 2000 e 2023, foram registrados 933.024 óbitos entre adultos com 25 anos ou mais nos quais hipertensão e infarto agudo do miocárdio foram registrados concomitantemente. A taxa ajustada por idade diminuiu de 19,84 para 16,26 por 100 mil habitantes, mas permaneceu mais elevada entre homens, pessoas negras não hispânicas e residentes do Sul e de áreas rurais^19^. Em um hospital terciário de Karachi, Paquistão, foram analisadas 14.601 internações por infarto agudo do miocárdio entre 1988 e 2018. Nos períodos 1988-1997 e 2008-2018, a idade mediana aumentou de 58 para 63 anos, a prevalência de hipertensão passou de 44% para 71% e a de diabetes de 38% para 53%, enquanto a mortalidade hospitalar diminuiu de 14% para 9%^20^.

No presente estudo, o número anual de óbitos permaneceu relativamente estável, com aumento de 3,8% entre 2015 e 2024, enquanto as taxas padronizadas por idade apresentaram redução. Além disso, 75,9% dos óbitos ocorreram entre pessoas com 60 anos ou mais. Em conjunto, esses resultados são compatíveis com a contribuição do envelhecimento populacional para a manutenção da elevada carga absoluta de mortalidade por infarto agudo do miocárdio e reforçam a necessidade de estratégias voltadas ao envelhecimento saudável, ao manejo de multimorbidades e à continuidade do cuidado^8^^,^^9^.

No presente estudo, as taxas regionais padronizadas pela população brasileira variaram de 96,58 por 100 mil habitantes no Sul a 120,97 por 100 mil no Nordeste. A persistência dessa variação após a padronização indica que as diferenças regionais não se devem exclusivamente à estrutura etária. Em revisão sistemática e metanálise de 37 estudos internacionais publicados até janeiro de 2024, que incluíram 21,1 milhões de pacientes com infarto agudo do miocárdio, a mortalidade no seguimento foi maior entre residentes de áreas rurais do que entre residentes de áreas urbanas (15,5% versus 13,4%; OR 1,18; IC95% 1,13; 1,24)^21^. Nos Estados Unidos, raça/cor, local de residência, cobertura por seguro, renda ou pobreza e escolaridade foram associados à mortalidade ou à readmissão após infarto agudo do miocárdio^22^, enquanto mulheres e minorias raciais apresentaram piores desfechos e diferenças no manejo da doença^23^. Em 40 coortes e registros dos Estados Unidos, Reino Unido e Canadá, que reuniram 14,2 milhões de pacientes com síndrome coronariana aguda, a mortalidade após infarto com supradesnivelamento do segmento ST foi maior entre pessoas negras do que entre pessoas brancas; entre pessoas asiáticas, a maior mortalidade foi identificada nas análises realizadas nos Estados Unidos^24^.

O predomínio da mortalidade entre homens é consistente com a literatura e pode estar relacionado a maior exposição a fatores de risco, menor utilização de serviços preventivos e diferenças comportamentais^2^. Entretanto, a análise das desigualdades por sexo deve considerar também especificidades na apresentação clínica e no acesso ao cuidado entre mulheres, que podem influenciar os desfechos em saúde. Além de fatores comportamentais, diferenças relacionadas ao diagnóstico, apresentação clínica e acesso ao tratamento têm sido apontadas como possíveis determinantes das disparidades observadas entre homens e mulheres após eventos coronarianos agudos^23^. Estratégias de prevenção devem, portanto, considerar essas diferenças, promovendo ações direcionadas a distintos perfis populacionais.

No presente estudo, as oscilações do número de óbitos entre 2020 e 2022 coincidiram com o período pandêmico, embora o delineamento empregado não permita atribuí-las causalmente à covid-19. Na Itália, durante uma semana da fase inicial da pandemia em 2020, as internações por infarto agudo do miocárdio foram 48% menores do que na semana correspondente de 2019; no norte da Califórnia, as taxas semanais de hospitalização diminuíram até 48% nas primeiras semanas da pandemia^10^^,^^11^. Em 32 estudos realizados em diferentes países, 19.140 pacientes com infarto com supradesnivelamento do segmento ST foram submetidos à intervenção coronariana percutânea primária durante a pandemia, em comparação com 68.662 antes da pandemia; o tempo porta-balão aumentou, em média, 8,10 minutos (IC95% 3,90; 12,30), e a mortalidade hospitalar foi maior (OR 1,27; IC95% 1,09; 1,49)^12^^,^^13^. Em metanálise de 26 estudos realizados em diferentes países, que incluíram 1.175.537 pacientes com covid-19, o infarto do miocárdio associou-se a maior mortalidade (efeito combinado 1,20; IC95% 1,09; 1,32)^25^. Nos Estados Unidos, em 2020, 5.818 das 555.540 internações por infarto apresentaram covid-19 concomitante; nesses casos, foram observadas menor realização de angiografia coronariana, maior frequência de complicações cardíacas e maior mortalidade hospitalar^26^. Esses resultados oferecem plausibilidade assistencial e clínica para interpretar as flutuações observadas na presente série temporal, sem permitir o estabelecimento de relação causal.

Os resultados deste estudo indicam que o enfrentamento da mortalidade por infarto agudo do miocárdio requer ações integradas nos diferentes níveis de atenção. Em 18 ensaios randomizados e por conglomerados, publicados entre 2003 e 2024 e realizados em 13 países, com 438.285 pessoas com doenças cardiovasculares atendidas na atenção primária ou hospitalar, intervenções de melhoria da qualidade aumentaram a prescrição de medicamentos hipolipemiantes (OR 1,46; IC95% 1,08; 1,99) e reduziram eventos cardiovasculares maiores (OR 0,84; IC95% 0,71; 0,98) e a mortalidade total (OR 0,87; IC95% 0,77; 0,98)^27^. Em metanálise de 14 ensaios clínicos randomizados, predominantemente realizados na China, com 1.463 pacientes com doença coronariana submetidos à intervenção coronária percutânea, intervenções estruturadas de cuidado contínuo de enfermagem após a alta melhoraram a qualidade de vida, a função cardíaca e a adesão ao tratamento, além de reduzirem os eventos cardiovasculares adversos (OR 0,18; IC95% 0,09; 0,36)^28^. A redução das desigualdades regionais deve ser considerada prioridade na formulação de políticas públicas, visando à construção de uma rede de cuidado capaz de responder à elevada carga das doenças cardiovasculares no país^29^^,^^30^.

Este estudo apresenta limitações inerentes ao uso de dados secundários, incluindo possíveis erros de classificação e incompletude de variáveis, especialmente aquelas de natureza socioeconômica. Embora a qualidade das informações tenha sido elevada para variáveis essenciais, a presença de dados ignorados pode introduzir viés de informação. Adicionalmente, a análise baseada em dados agregados limita a realização de inferências em nível individual, restringindo a avaliação de fatores clínicos e contextuais associados à mortalidade.

Em síntese, observou-se elevada carga de mortalidade por infarto agudo do miocárdio ao longo do período analisado, com predomínio em idosos, maior ocorrência em homens e desigualdades regionais persistentes. A redução das taxas padronizadas por idade indica declínio da mortalidade ajustada, apesar da estabilidade do número absoluto de óbitos. Esses achados reforçam a necessidade de fortalecimento das ações de prevenção, diagnóstico oportuno e organização da rede de atenção às doenças cardiovasculares no Sistema Único de Saúde, com foco na redução das desigualdades e na preparação do sistema de saúde para o impacto crescente do envelhecimento populacional sobre a carga cardiovascular nas próximas décadas^8^^,^^9^. 

## Data Availability

O banco de dados consolidado utilizado na análise está disponível em repositório público: https://doi.org/10.5281/zenodo.19488974[xref ref-type="bibr" rid="r15"]^15^[/xref].

## Material suplementar

Supplementary Material Material suplementar
